# West Nile Virus Mosquito Vectors (Diptera: Culicidae) in Germany

**DOI:** 10.3390/v12050493

**Published:** 2020-04-28

**Authors:** Helge Kampen, Cora M. Holicki, Ute Ziegler, Martin H. Groschup, Birke Andrea Tews, Doreen Werner

**Affiliations:** 1 Friedrich-Loeffler-Institut, Federal Research Institute for Animal Health, Institute of Infectology, 17493 Greifswald-Insel Riems, Germany; 2 Friedrich-Loeffler-Institut, Federal Research Institute for Animal Health, Institute of Novel and Emerging Infectious Diseases, 17493 Greifswald-Insel Riems, Germany; 3 Leibniz-Centre for Agricultural Landscape Research, 15374 Muencheberg, Germany

**Keywords:** *Culex pipiens*, first report, Germany, mosquito vectors, overwintering, transmission, West Nile virus

## Abstract

In 2018, West Nile virus (WNV) broke out for the first time in Germany, with continuation of the epidemic in 2019, involving birds, horses and humans. To identify vectors and characterize the virus, mosquitoes were collected in both years in zoological gardens and on a horse meadow immediately following the diagnosis of disease cases in birds and horses. Mosquitoes were identified and screened for WNV by qRT-PCR, with virus-positive samples being sequenced for the viral envelope protein gene. While no positive mosquitoes were found in 2018, seven mosquito pools tested positive for WNV in 2019 in the Tierpark (Wildlife Park) Berlin. The pools consisted of *Cx. pipiens* biotype *pipiens* (*n* = 5), and a mixture of *Cx. p.* biotype *pipiens* and *Cx. p.* biotype *molestus* (*n* = 2), or hybrids of these, and were collected between 13 August and 24 September 2019. The virus strain turned out to be nearly identical to two WNV strains isolated from birds diseased in 2018 in eastern Germany. The findings represent the first demonstration of WNV in mosquitoes in Germany and include the possibility of local overwintering of the virus.

## 1. Introduction

West Nile virus (WNV) is an arbovirus of the family Flaviviridae which naturally circulates between birds and mosquitoes. Birds show very different mortality rates depending on species [1]. The virus can also be transmitted to humans and horses, but these are considered dead end hosts. Humans can develop West Nile fever (WNF) or West Nile Neuroinvasive Disease (WNND). Most infections in humans remain asymptomatic, but 20% develop mild flu-like symptoms, occasionally accompanied by fever. In 1% of the cases, predominantly elderly people, a neuroinvasive disease such as meningoencephalitis may develop, sometimes leading to the death of the patient [2]. Horses may also show neurological disorders with considerable mortality rates [3].

After its detection in Uganda in 1937, WNV has been detected in numerous countries and is now considered to occur on all continents, except Antarctica [4]. In Europe, first evidence of circulation was obtained serologically from two Albanian citizens in 1958 [5], while the virus was isolated not before 1963 from mosquitoes collected in French Camargue [6]. Migratory birds in which the virus may persist for months are thought to be natural vehicles of the disease agent, regularly introducing it into Europe from endemic areas in Africa on their way to the north in spring [7].

WNF cases and geographically defined outbreaks have been observed in southern Europe for decades, affecting both horses and humans [7,8]. While these had mainly been caused by moderately pathogenic lineage 1 virus types, the majority of more recent virus isolations in Europe was characterized by a higher pathogenicity and associated with more fatal disease cases. Viruses isolated belonged to WNV lineage 1 Israeli/American cluster and lineage 2 [7,9]. Correspondingly, mortality rates were comparably high during outbreaks in the last two decades, such as in Greece, Italy and Romania [10,11,12].

In 2018, Europe experienced its most serious WNF epidemic ever, by far exceeding the sum of case numbers (human and equine) of the seven years before: more than 2,000 people were diagnosed infected and 180 died [13]. Moreover, more countries were affected than during previous transmission seasons [14].

WNV has been isolated from, or detected in, field-collected specimens of numerous mosquito species native to Europe [15], and experimental transmission has been demonstrated in *Aedes albopictus, Ae. caspius, Ae. detritus, Ae. dorsalis, Ae. geniculatus, Ae. japonicus, Ae. punctor, Ae. vexans, An. plumbeus, Culex modestus, Cx. pipiens, Cx. theileri, Cx. torrentium* and *Uranotaenia unguiculata* [16,17,18]. Among these, *Culex* species, in particular *Cx. pipiens*, are considered the most important vectors [19,20,21,22,23], although in some experimental studies the invasive Asian bush mosquito *Ae. japonicus*, which has recently spread over considerable parts of Central Europe [24], has proven even more efficient than *Cx. pipiens* [25,26]. Despite being a moderate WNV vector in the laboratory [25], *Ae. vexans* may also be epidemiologically important just due to its generally high prevalence and abundance.

Overwintering of WNV takes place in infected mosquito females [27,28,29,30], and vertical transmission in mosquitoes is possible, although apparently not efficient [25,31,32]. 

Germany was one of the countries in which WNF broke out for the first time in 2018, probably supported by an extraordinarily hot weather period in late summer and early autumn [33]. In that year, 12 birds and 2 horses were found infected [34]. The epidemic continued as early as July 2019, suggesting that the virus overwintered in native mosquitoes [34]. To date, dozens of birds and horses, as well as five diseased humans, have tested positive [35]. The cases concentrated on the eastern part of Germany, where warm weather conditions prevailed. 

Prior to 2018, no evidence had been found on the circulation of WNV in Germany, although great numbers of animal and human sera as well as field-collected mosquitoes were tested for flavivirus (including WNV) infection, over several years [36,37,38,39,40,41,42]. 

We here describe mosquito examinations linked to 2018 and 2019 WNF outbreak scenarios in eastern Germany, with the first detection of WNV in mosquitoes collected in Germany. In addition, we present geographical distribution data accumulated over several years for major WNV mosquito vector species for Germany.

## 2. Materials and Methods

### 2.1. Mosquito Collection and Identification

Monitoring linked to WNF cases: After WNV infection had been diagnosed in zoo birds and a horse, EVS (encephalitis virus surveillance) traps (BioQuip, Products, Rancho Dominguez, CA, USA), equipped with dry ice as a CO_2_ attractant, were operated continuously in Halle, federal state of Saxony-Anhalt (zoo, 31 August to 22 September 2018, starting three days after diagnosis of a WNV case in a great grey owl; location 1 in Figure S1); in Poing, Bavaria (wildlife park, 17 to 30 September 2018, starting five days after diagnosis of a WNV case in a great grey owl; location 2 in Figure S1); in Kahla, Brandenburg (horse pasture, 1 to 11 October 2018, starting 10 days after diagnosis of a WNV case in a foal; location 3 in Figure S1); and in Berlin (wildlife park (Tierpark), 6 to 29 September 2018, starting two days after preliminary diagnosis of WNV infection in the first of two deceased snowy owls, and 12 September to 6 October 2019, three weeks after diagnosis of a WNV case in a snowy owl; location 4 in Figure S1). For logistical reasons, in Halle, Poing and Kahla, no more than 10 EVS traps each could be operated by local attendants, while the Tierpark Berlin was sampled by 20 EVS traps per year, taken care of by the authors themselves. In the zoos/wildlife parks, the traps were evenly distributed over the complete park area, while on the horse pasture, they were placed along two vegetated fence lines (see Table S1 for geocoordinates of trapping sites). All traps were hung into trees and bushes, right next to animal enclosures (including aviaries), at a height of about 1.8 m.

An additional BG-Sentinel trap equipped with a gas tank as CO_2_ source was operated in the Tierpark Berlin for 24 hrs a week, from 26 July to 9 September and from 7 October to 23 November 2019, and continuously during the EVS trapping period (12 September to 6 October 2019).

All traps were checked in the morning (09:00–12:00 o’clock), with mosquito collection nets being replaced and evaporated dry ice replenished. Used nets containing mosquitoes were immediately put on dry ice to kill and store collected mosquitoes. Depending on the monitoring site, these were either brought into the laboratory and further processed on the same day, or kept on dry ice overnight and then transferred to plastic vials, which again were put on dry ice until further processing in the laboratory.

Mosquitoes were morphologically identified by species or species complex on a chilling table under a stereomicroscope, using the determination keys by Schaffner et al. [43] and Becker et al. [44]. Specimens belonging to the same species/species complex and sex collected on the same day and at the same sampling site were pooled, with up to 10 individuals per pool. Due to low numbers, all males were processed individually. Pools and single individuals were stored in a ‒80 °C freezer until nucleic acid extraction.

Mosquito pools testing positive in the subsequent virus-PCR assays and consisting of *Culex pipiens* complex species were retroactively subjected to genetic species and biotype differentiation. For this purpose, a multiplex real-time PCR was used, simultaneously targeting the *ace2* gene for separating *Cx. torrentium* from other species of the *Cx. pipiens* complex, and the microsatellite locus CQ11 for differentiating the *Cx. pipiens* biotypes [45].

General Germany-wide monitoring: Mosquito monitoring, consisting of trapping by both BG-Sentinels (Biogents, Germany) and EVS traps, larval sampling, and the citizen science project ‘Mueckenatlas’ (mosquito atlas [46]) have all been carried out throughout Germany since 2011 (trapping, larval sampling) or 2012 (‘Mueckenatlas’), respectively, including thousands of collection sites [47,48] to map mosquito occurrence and distribution in Germany. Traps were equipped with CO_2_ gas tanks and run from April to October for 24 hrs a week. BG-Sentinels were usually operated at one and the same site for at least one mosquito season, but sometimes for up to three years, while EVS traps were used more flexibly and short-termed, with collection periods of several weeks per season up to a complete mosquito season at a given site. Most of the collection sites (particularly BG-Sentinel sites) were selected randomly, partly supported by computer algorithms, covering as many landscape structures as possible, but some (EVS trapping sites) were sampled deliberately, based on previous information on particularly high local mosquito density or species diversity, or the occurrence of extraordinary (e.g., invasive or very rare) species.

Determination of collected specimens was performed morphologically as described above, although not necessarily with cooling. In the case of damaged specimens and species belonging to species complexes, genetic identification was conducted according to Heym et al. [49].

All monitoring data were entered into the German mosquito database CULBASE, from which they were extracted for the purpose of mapping potential WNV vectors.

### 2.2. Sample Homogenization and Nucleic Acid Extraction

For RNA and DNA extraction, mosquito pools were processed as described by Scheuch et al. [40]. Briefly, samples were homogenized with three 3 mm steel beads in 450 µL (for single mosquitoes) or 750 µL (pools with more than one specimen) serum-free minimal essential medium, supplemented with penicillin, streptomycin, gentamicin and amphotericin B (ThermoFisher Scientific, MA, USA), for 2 min at 30 Hz in a Tissue Lyzer II (Qiagen, Hilden, Germany). Subsequently, debris was pelleted by centrifugation for 3 min at 20,000 g. 200 µL of the supernatant was heat-inactivated (10 min, 70 °C) before simultaneous DNA/RNA extraction according to the manufacturer’s instructions, using the NucleoMag Vet Kit (Macherey-Nagel, Düren, Germany) on a BioSprint 96 workstation (Qiagen). Extracted nucleic acids were used for RT-qPCR virus analysis, RT-PCR and sequencing, as well as for genetic mosquito species identification and biotype differentiation.

### 2.3. Nucleic Acid Amplification

Extracted nucleic acid solutions were tested for the presence of WNV-RNA using two published RT-qPCRs [50], one targeting the 5’-untranslated region (INEID) and one the nonstructural NS2A protein gene region (WNF-FLI). Samples were also tested for Usutu virus (USUV) using a USUV-specific RT-qPCR, targeting a nonstructural protein 1 gene region [51]. For all RT-qPCRs, either the AgPath-ID One-Step RT-PCR Kit (ThermoFisher Scientic) or the QuantiTect Probe RT-PCR Kit (Qiagen) was used. Realtime PCRs were run on a CFX96 RealTime System (Bio-Rad, Munich, Germany).

Nucleic acids extracted from dilution series of WNV strains 203204 (GenBank accession number: JF719066), 204913 (KF114267), NY99 (AF196835) and Austria 361/10 (HM015884), and from USUV strain Europa 3 (HE599647), were used as positive controls.

### 2.4. E Gene Analysis

For all positive mosquito pools, the complete viral envelope protein coding sequence (E gene) was determined. The corresponding RNA region was amplified in two parts through one-step RT-PCR by means of the Super Script III One Step RT-PCR Kit (ThermoFisher Scientific), using primers BT1224/BT1227 and BT1228/BT1230 (Table S2). Amplicons were sequenced using the Big Dye Terminator v1.1 Cycle sequencing Kit (Applied Biosystems, CA, USA) and the primers listed in Table S1, and analyzed on a 3130 Genetic Analyzer (Applied Biosystems). Resulting nucleotide sequences were trimmed (i.e., primer sequences removed) and combined to yield the E gene sequence of the different samples.

### 2.5. Cell Culture and Virus Isolation

Vero cells (L 0015, Collection of Cell Lines in Veterinary Medicine (CCLV), Friedrich-Loeffler-Institut (FLI), Insel Riems, Greifswald, Germany) and C6/36 cells (L 1299, CCLV, FLI, Insel Riems, Greifswald, Germany) were used to isolate virus from mosquito samples. Vero cells were routinely kept in MEM with Hank’s and Earle’s salts, non-essential amino acids and 10% fetal calf serum (FCS) at 37 °C and 5% CO_2_. C6/36 cells were routinely kept in Schneider’s insect medium with 10% FCS at 28 °C without CO_2_. All media were obtained from the CCLV and were supplemented with penicillin and streptomycin (Gibco/ThermoFisher Scientific, MA, USA).

Virus isolation was attempted on Vero and C6/36 cells in parallel. Cells were seeded in 96-well plates, 24 h later inoculated with 50 µL of RT-PCR positive mosquito homogenate and incubated at their respective temperatures. A day later, the medium was replaced by fresh medium, and two days after infection, cells were transferred to 24-well plates to be co-cultured with naïve cells. These were incubated for 4–6 days and checked regularly for cytopathogenic effect. The supernatant of those cultures was tested for virus by RT-qPCR as described above. Samples with Ct values below 24 were used to infect C6/36 and Vero cells in T75 cell culture flasks to generate virus stocks.

## 3. Results

### 3.1. Identification of WNV Vectors

A total of 3,103 mosquitoes of eight culicid taxa (species, species complexes and groups) were trapped in the sampled WNV outbreak areas; 862 in 2018 and 2,241 in 2019 (Table 1). The majority of these, about 92% in 2018 and 96% in 2019, belonged to the *Cx. pipiens* complex. *Culiseta annulata* (1.7%), *Aedes vexans* (1.5%) and members of the *An. maculipennis* complex (1.3%) were collected in the tens, other species only as occasional individuals.

All mosquitoes, combined to 445 pools (pooled according to species, sex, collection date and site), were subjected to PCR screening for WNV and USUV. Of these, seven pools collected in 2019 in the Tierpark Berlin tested positive for WNV-RNA with both PCRs (Table 2). No pool or individual was positive in 2018, and no pool or individual was positive with only one of the two PCR assays in 2019. All samples were negative for USUV. Of the WNV-positive pools, six consisted of 10 *Cx. pipiens* complex individuals, and one consisted of 6 *Cx. pipiens* complex individuals. All positive pools contained female mosquitoes. Subsequent genetic species differentiation produced signals for *Cx. pipiens* biotype *pipiens* in five pools, and for both *Cx. pipiens* biotype *pipiens* and *Cx. pipiens* biotype *molestus* in two pools (Table 2).

**Figure 1:**
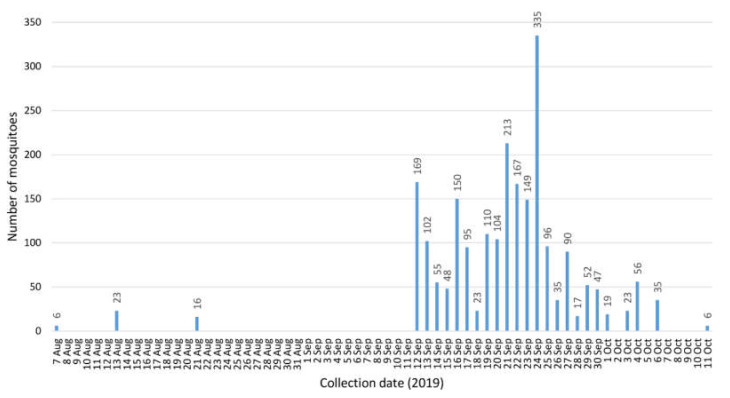
Mosquito numbers collected over time in the Tierpark Berlin in 2019 (26 Jul–9 Sep and 7 Oct–23 Nov: collection by one BG-Sentinel alone; 12 Sep–6 Oct: collection by 20 EVS traps plus one BG-Sentinel; except for 10 and 11 Sep trapping was performed continuously).

The positive mosquitoes were collected 13 August, and 12, 13 (2 pools), 17 and 24 (2 pools) September 2019 (Table 2). A first dead snowy owl had been found on 4 August 2019 and confirmed WNV-positive on 20 August 2019. In total, 33 dead birds had been collected in Berlin during 2019 [35], confirming the hot spot situation. In that year, the BG-Sentinel trap alone collected three times during its operation period before 12 September, and once after 6 October (Figure 1). After 11 October, no more mosquitoes were caught. Not surprisingly, when both the BG-Sentinel and the 20 EVS traps were continuously active, numbers of captured mosquitoes increased considerably. Thus, the majority of mosquito specimens were collected from 12 September to 6 October (Figure 1). Minimum infection rates (MIR) per 1000 and infection rated per maximum likelihood estimates were calculated using the Excel add-in of the Center for Disease Control and Prevention (https://www.cdc.gov/westnile/resourcepages/mosqSurvSoft.html). Only female mosquitoes of the *Cx. pipiens* complex were included in the calculation (Table 3). Overall MIR for the complete collection period was 3.29. On the positive days, it accounted to 50 (13 Aug), 6.85 (12 Sep), 21.05 (13 Sep), 11.49 (17 Sep) and 6.10 (24 Sep). 

### 3.2. Viral Analyses

The viral E gene nucleotide sequences (1503 nt) clearly show all RT-qPCR positive pools to contain virus belonging to WNV lineage 2. Sequences were identical in five of the seven samples. A comparison of the samples shows that those collected on 13 September 2019 contain two and three substitutions, respectively (positions 1050, 1384 and 2010; numbering corresponds to full length genomes), with the change at position 1384 (present only in sequence MN921232) leading to an amino acid substitution, while the remaining two substitutions are silent. Nucleotide sequences have been deposited in GenBank (see Table 2 for accession numbers). Comparison with corresponding WNV sequences of German bird isolates from 2018 (MH924836, MH986055 and MH986056, which have identical E gene sequences) revealed that the mosquito-derived samples differ in only one or two nucleotides each, again pertaining to positions 1050 (13 Sep, both sequences), 1384 (13 Sep, MN921232) and 2010 (all other samples). Thus, the 2019 mosquito isolates differ more from each other than from the 2018 bird isolates. Furthermore, comparison with 72 other recent European WNV lineage 2 isolates showed no more closely related sequences. The most similar sequence is from a Czech isolate from 2013 (KM203862) that differs in five and six nucleotides, respectively, from the 2019 mosquito-derived sequences. The Czech isolate has already been found to be the closest relative to the 2018 bird sequence in a full-length genome comparison [33]. We regard the E gene sequence analysis alone insufficient to build a well-founded phylogenetic tree, considering the paucity of mutations.

All seven positive mosquito pools were inoculated on Vero and C6/36 cells in parallel to isolate infectious virus. Recovery of infectious virus was first confirmed by RT-qPCR with cell culture Ct values lower than in the original inoculum and by production of cell culture viral stocks. Infectious virus could be obtained from three pools (13 Sep on Vero cells, 17 Sep and 24 Sep on C6/36 cells). Full-length sequences of viruses grown on C6/36 cells were determined by new generation sequencing (NGS) as part of an epidemiological analysis of 2018 and 2019 WNV isolates, presented in another study [35].

### 3.3. Occurrence of Major Potential WNV Vectors in Germany

**Figure 2:**
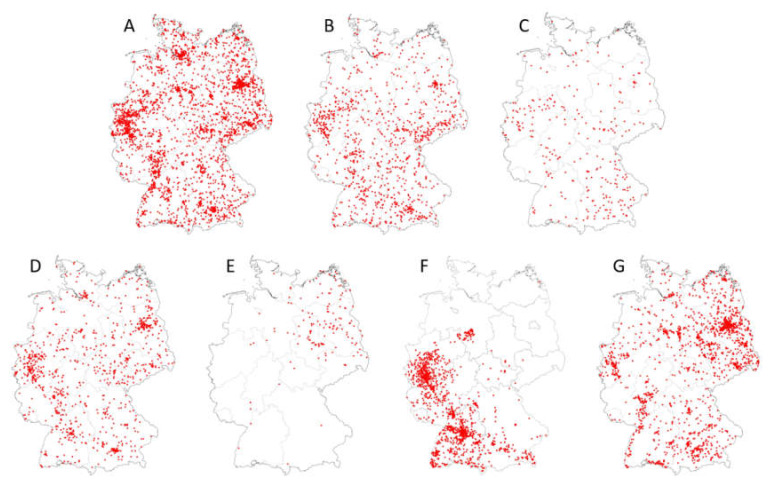
Distribution maps (presence data) of major potential WNV vectors in Germany (data extracted from CULBASE database, with collection dates from 2012 to 2018; red dots represent collections sites). (**A**) *Cx. pipiens* biotype *pipiens*, (**B**) *Cx. pipiens* biotype *molestus*, (**C**) *Cx. pipiens* biotype *pipiens* x *Cx. pipiens* biotype *molestus* hybrids, (**D**) *Cx. torrentium*, (**E**) *Cx. modestus*, (**F**) *Ae. japonicus*, (**G**) *Ae. vexans.* See [35] for areas of WNV detection in vertebrates in eastern Germany in 2018 and 2019.

Figure 2 displays the geographic distributions of *Cx. pipiens* complex species, biotypes and hybrids of biotypes (*Cx. pipiens* biotype *pipiens, Cx. pipiens* biotype *molestus, Cx. pipiens* biotype *pipiens* x biotype *molestus, Cx. torrentium*), as well as of *Cx. modestus, Ae. japonicus* and *Ae. vexans*, based on adult and larval collections throughout Germany of the years 2011 to 2018 (data are deposited in the German mosquito database CULBASE and are available on reasonable request). In addition to *Ae. albopictus*, which only occurs sporadically in Germany [52,53,54], these mosquito taxa are considered the most efficient potential WNV vectors in Central Europe. Although some of them, in particular *Cx. modestus* and *Ae. japonicus*, do not appear to be evenly distributed over the whole country, the maps suggest that potential WNV vectors probably do occur throughout Germany. Further, all of them are active throughout the vegetative season, including late summer (Table 4), which is the warmest period of the year and thus the period with the highest risk of biological WNV transmission in Germany.

## 4. Discussion

In 2018, WNV broke out for the first time in Germany. Virus isolates obtained from birds showed high degrees of sequence similarities among each other, and between these and a Central European subclade II strain of WNV lineage 2 isolated in the Czech Republic in 2013 [33]. However, the number of cases observed in 2018 remained limited, and no human case occurred. Screening of mosquitoes collected at outbreak sites yielded no positive mosquitoes. Even before, in summers 2016 and 2017, more than 3,800 mosquitoes collected in the Tierpark Berlin in the framework of another study and using different collection approaches tested negative for flaviviruses [55]. The number of mosquitoes collected in the present study was lower in 2018 than in 2019, and would have shown 1 positive mosquito in 1000 with a probability of around 58% only, but the likelihood of finding a positive sample with 2 mosquitoes in 1000 already accounted for over 80%. The overall minimum infection rate for 2019 was 3.29. A similar rate in 2018 would have been detected even with the smaller sample size, but a much lower infection rate was probably given due to the lower case number in birds. In 2019, the first WNV infection cases emerged much earlier in the season, diagnosed cases were far more numerous than in 2018, and infections in humans and horses could be detected in addition to avian infections [35]. This, combined with a hotspot situation in Berlin (particularly many infected wild and zoo birds through August and October 2019) [35,56] and a higher sampling effort at that location, is probably the reason why mosquito screening was successful, producing seven positive samples in the Tierpark Berlin, all of which consisted of *Cx. pipiens* complex specimens. At least three of the seven positive pools contained infectious WNV, as virus could be propagated in cell culture to yield viral stocks (pools from 13 Sep, 17 Sep and 24 Sep). Isolation was attempted on Vero and C6/36 cells in parallel, but it remains unclear why one of the three isolates could only be cultured on Vero, and two only on C6/36 cells. 

All mosquito samples were of sufficient quality to determine the E gene sequence and compare it to the 2018 bird isolates and other recent European (lineage 2) WNV strains. The most similar sequences found had been obtained from birds deceased in Germany in 2018, with only single nucleotide change. The second closest sequence also represented the second most similar to the 2018 bird-derived sequences, a Czech isolate from 2013. As compared to this, the 2019 mosquito sequences from Germany were characterized by one and two additional nucleotide differences from the 2018 bird-derived German sequences. This might suggest that the mosquito virus found in 2019 was derived from the WNV strain found in German birds in 2018 and point to overwintering of the virus in Germany.

Although USUV lineage Africa 2 was detected in birds in Berlin in 2017 and lineage Europe 3 in 2018 [42], none of the mosquitoes tested in the present study were positive for USUV. The obvious conclusion would be that virus prevalence in mosquitoes remained below detection limit in the study period.

While the mere finding of a virus in a completely homogenized haematophagous arthropod in the first place demonstrates feeding on an infectious blood source only, and does not allow conclusions about the vector status of this arthropod, members of the *Cx. pipiens* complex have been found positive for WNV in numerous field studies and shown to be competent WNV vectors in the laboratory [22]. Specifically, both biotypes of *Cx. pipiens* occurring in temperate climatic zones, biotype *pipiens* and biotype *molestus*, as well as *Cx. torrentium*, have proven vector-competent [18,23,57].

However, the complex species, biotypes and biotype hybrids differ in behavior and physiology, resulting in different vector capacities. While *Cx. torrentium* occurs throughout the vegetative season and is considered strictly ornithophilic [44], the *Cx. pipiens* biotypes have different seasonal activities, as well as different breeding site and blood host preferences. *Culex pipiens* biotype *molestus* is autogenous (i.e., does not need a blood meal for producing the first seasonal batch of eggs), active during the vegetative season only, commonly breeds in artificial containers in dark and humid places, and has a preference for mammals, particularly humans, as blood hosts, although being relatively opportunistic in host choice. By contrast, *Cx. pipiens* biotype *pipiens* is characterized by anautogeny (i.e., needing a blood meal even for the first batch of eggs), breeding in almost any kind of natural and man-made water pools, activity throughout the year and a high degree of ornithophily [44,58].

While *Cx. torrentium* and *Cx. pipiens* biotype *pipiens* probably mainly circulate viruses, such as WNV, between birds, due to their ornithophilic behavior, and *Cx. pipiens* biotype *molestus* occasionally contributes to transmitting viruses between birds and mammals [44,59], the hybrids of the two *Cx. pipiens* biotypes which indiscriminately bite both birds and mammals represent excellent bridge vectors and are therefore thought to be major actors in WNV transmission, although final evidence is still lacking [59,60,61]. Data on the occurrence of such hybrids are rare [62], but a preliminary study from Germany showing the presence of hybrids at three sites suggested crossbreeding between the biotypes to be a frequent event [45]. Confirming this assumption, our data demonstrate a wide geographic distribution of hybrids as well as an extended seasonal occurrence (mid-April to mid-October). It has to remain unanswered, however, whether the two WNV-positive mosquito pools producing signals for both *Cx. pipiens* biotype *pipiens* and *Cx. pipiens* biotype *molestus* in this study contained hybrids, or must be attributed to a mixture of the two biotypes. 

Based on prevalence and abundance, in particular in human settlements, as well as biting behavior and vector competence, the conclusions drawn by Kilpatrick et al. [63] for the US—that the risk of WNV transmission posed by *Cx. pipiens* is much higher than for any other vector-competent culicid species present—may be transferred to Germany. Taken together, though, other mosquito species may also significantly contribute to WNV epidemiology, due to being highly prevalent and abundant (e.g., *Ae. vexans*) or highly vector-competent (e.g., *Ae. japonicus, Ae. albopictus*).

## 5. Conclusions

E gene sequence analysis clearly shows that the 2018 WNV virus strain continued to circulate in Germany in 2019. The finding of the first WNV-positive mosquito sample in mid-August 2019, together with the early seasonal start of disease epidemiology (4 August as the date of death of the first bird diagnosed with WNV infection in 2019 in the Tierpark Berlin), also strongly argue for the virus to have overwintered in Germany, most likely in hibernating *Cx. pipiens* mosquito females. It must be assumed that the 2019 WNV epidemiology produced a much greater viral distribution at the end of the mosquito season than in 2018, facilitating an even more threatening situation at the onset of the 2020 mosquito season, repeated overwintering of the virus provided. Given a similarly hot summer as in previous years, a further increase in WNV cases must be expected for 2020.

## Figures and Tables

**Table 1:** Mosquitoes collected at the four collection sites in 2018 and 2019.

Mosquito Taxon	2018			2019		
	No. Specimens Tested	No. Pools Tested	No. Pools WNV-Positive	No. Specimens Tested	No. Pools Tested	No. Pools WNV-Positive
Ae. cinereus/geminus *	1	1	0	---	---	---
*Ae. japonicus*	1	1	0	---	---	---
*Ae. vexans*	19	15	0	27	10	0
*An. claviger*	2	2	0	3	3	0
*An. maculipennis* complex	6	6	0	33	14	0
*An. plumbeus*	1	1	0	6	5	0
*Cs. annulata*	36	19	0	16 (incl. 1 male)	9	0
*Cx. pipiens* complex	796 (incl. 2 males)	94	0	2154 (incl. 25 males)	263	7
Not identifiable	---	---	0	2	2	0
total	862	139	0	2241	306	7

* Females not reliably distinguishable.

**Table 2:** Collection dates and Ct values of mosquitoes from the Tierpark Berlin belonging to the *Culex pipiens* complex that tested positive in two WNV-specific qRT-PCR assays (FLI-WNF, INEID; names of assays refer to primer names in the original paper [50]).

Collection Date	No. of Mosquitoes/ Pool	Mosquito Taxon	Ct FLI-WNF	Ct INEID	Virus Isolation	E gene Sequence GenBank Accession No.
08-13-2019	6	*Cx. pipiens* biotype *pipiens*	22.10	26.88	not successful	MN921236
09-12-2019	10	*Cx. pipiens* biotype *pipiens*	21.94	25.62	not successful	MN921230
09-13-2019	10	*Cx. pipiens* biotype *pipiens*	23.34	27.45	successful on Vero cells	MN921231
09-13-2019	10	*Cx. pipiens* biotype *pipiens*	31.15	36.81	not successful	MN921232
09-17-2019	10	*Cx. pipiens* biotype *pipiens* x biotype *molestus*	29.15	33.53	successful on C6/36 cells	MN921233
09-24-2019	10	*Cx. pipiens* biotype *pipiens* x biotype *molestus*	22.18	26.09	successful on C6/36 cells	MN921234
09-24-2019	10	*Cx. pipiens* biotype *pipiens*	29.46	33.12	not successful	MN921235

**Table 3:** Minimum infection rates (MIR) and infection rated per maximum likelihood estimate (IR MLE) for *Cx. pipiens* complex females collected in the Tierpark Berlin in 2019.

	No. Pools Positive/Tested	No. Individuals Tested	MIR (95% CI)	IR MLE (95% CI)
2019	7/237	2128	3.29 (0.86–5.72)	3.33 (1.47–6.56)
08-13-2019	1/7	20	50.00 (0.00–145.52)	52.12 (3.07–245.89)
09-12-2019	1/15	146	6.85 (0.00–20.23)	6.85 (0.40–33.23)
09-13-2019	2/10	95	21.05 (0.00–49.92)	22.17 (4.07–73.06)
09-17-2019	1/9	87	11.49 (0.00–33.89)	11.49 (0.68–56.05)
09-24-2019	2/34	328	6.10 (0.00–14.52)	6.18 (1.11–20.21)

**Table 4:** Seasonal activity of major WNV vector taxa in Germany, based on trap collections of females from April to October 2011 to 2018 (24 h per week).

Mosquito Taxon	Seasonal Collection	
	First Calendar Week	Last Calendar Week
*Ae. japonicus*	14	41
*Cx. modestus*	13	41
*Cx. pipiens* biotype *pipiens*	13	49
*Cx. pipiens* biotype *molestus*	13	41
*Cx. pipiens* biotype *pipiens* x biotype *molestus* (hybrids)	16	42
*Cx. torrentium*	13	42

## Supplementary Materials

The following are available online at https://www.mdpi.com/1999-4915/12/5/493/s1, Figure S1: Geographic locations of sampling sites in 2018 and 2019 with mosquitoes screened for WNV (1: Zoo Halle, 2: Wildlife Park Poing, 3: horse pasture Kahla, 4: Tierpark Berlin), Table S1: Primers used for amplification and sequencing of the E gene, Table S2: Geocoordinates of mosquito trapping sites.
