# Supplementary material for: West Nile Virus Mosquito Vectors (Diptera: Culicidae) in Germany
Source: Viruses. 2020 Apr 28;12(5):493. doi: 10.3390/v12050493 (PMC7290393; doi:10.3390/v12050493)
Supplement: Supplementary file 1 [file viruses-12-00493-s001.pdf]

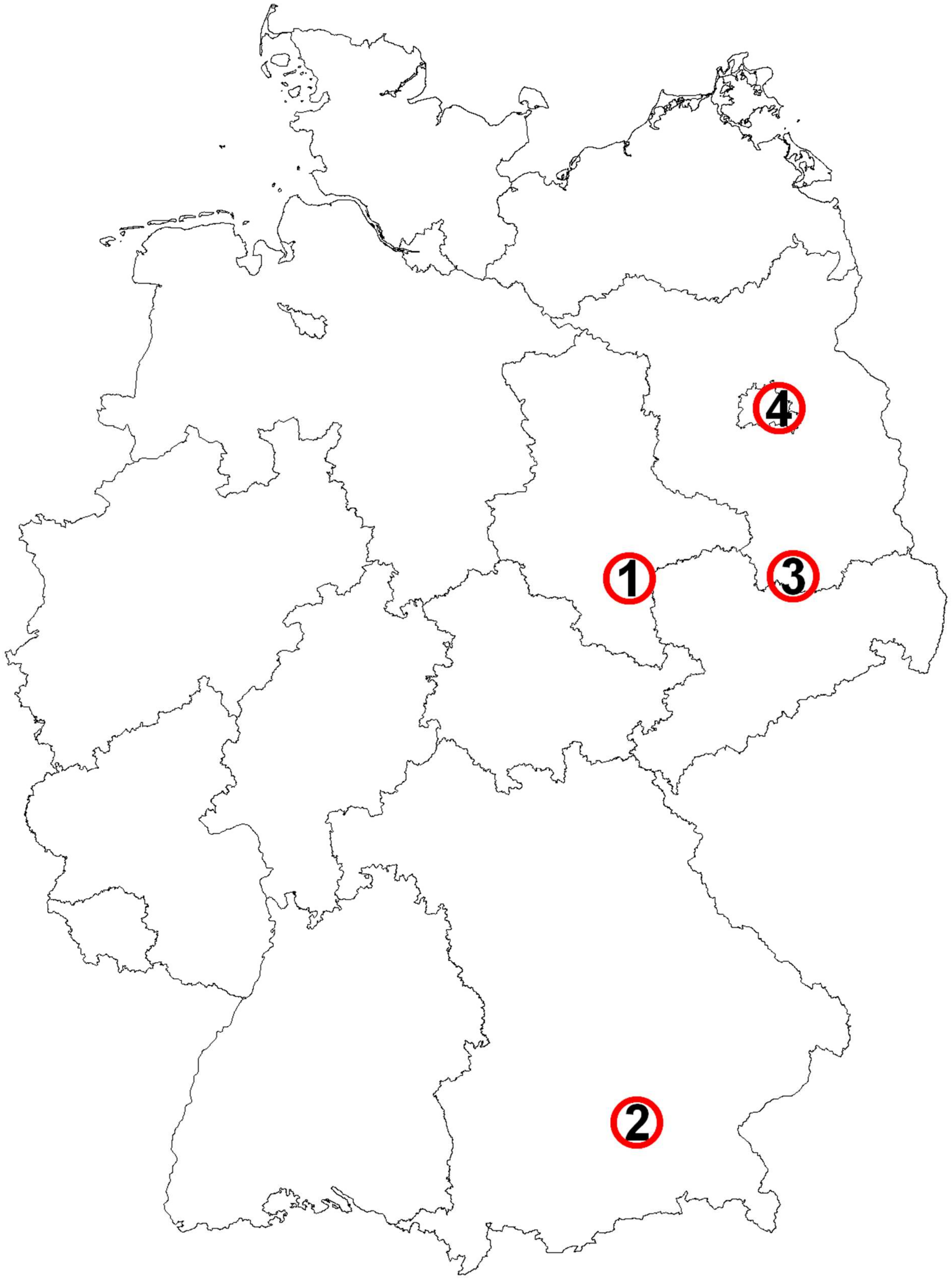

Figure S1. Geographic locations of sampling sites in 2018 and 2019 with mosquitoes screened for WNV (1: Zoo Halle, 2: Wildlife Park Poing, 3: horse pasture Kahla, 4: Tierpark Berlin)

Table S1. Geocoordinates of mosquito trapping sites.

Location	No. according to Figure S1	Year	Trap type	Geocoordinates of trapping sites
Halle (Zoo)	1	2018	EVS	51.505318, 11.963355
				51.504670, 11.962963
				51.504654, 11.961558
				51.505098, 11.960785
				51.505679, 11.961032
				51.506033, 11.961317
				51.506260, 11.962287
				51.505531, 11.962003
				51.504857, 11.961907
				51.505378, 11.963216
Poing (Wildlife Park)	2	2018	EVS	48.174844, 11.831776
				48.173813, 11.831669
				48.174579, 11.832892
				48.174343, 11.833793
				48.174064, 11.835070
				48.174529, 11.837409
				48.172561, 11.835725
				48.172533, 11.833718
				48.172340, 11.837388
				48.172526, 11.829255
Kahla (horse pasture)	3	2018	EVS	51.463674, 13.562991
				51.463587, 13.563602
				51.463448, 13.564369
				51.463368, 13.564884
				51.463050, 13.564889
				51.469494, 13.559809
				51.469381, 13.560372
				51.469264, 13.561175
				51.469204, 13.562078
				51.469187, 13.563033
Berlin (Tierpark)	4	2018, 2019	EVS	52.497853, 13.530766
				52.499250, 13.535497
				52.499766, 13.540551
				52.501758, 13.536366
				52.503652, 13.535234
				52.502777, 13.535733
				52.502568, 13.531656
				52.504541, 13.534102
				52.504201, 13.533180
				52.504305, 13.531774
				52.507930, 13.528534
				52.506114, 13.531528
				52.507571, 13.526442
				52.507353, 13.535827
				52.504901, 13.534408
				52.505744, 13.534108
				52.507216, 13.533260

			52.508238, 13.533191
			52.502738, 13.525686
			52.498075, 13.526565
	2019	BG	52.506565, 13.531903

Table S2. Primers used for amplification and sequencing of the E gene.

Primer	Sequence (5′-3′)	Reference
R1300	TTGGCGCATGTGTCAATGCT	[1]
BT1224	GCCTGCCAATATCAATGGCAC	this study
BT1225	TGACCGTGAATCCATTTGTGTC	this study
BT1226	ACGGGGGTTCGAGTTCAATC	this study
BT1227	CCACGTGGAGGAATCGGG	this study
BT1228	TCCTGCGACCCTAGAGCC	this study
BT1229	AGAGGATGGGGAAATGGCTG	this study
BT1230	AGGAAGCAACACAATGCAACGC	this study

References

1. Grinev, A.; Daniel, S.; Stramer, S.; Rossmann, S.; Caglioti, S.; Rios, M. Genetic variability of West Nile virus in US blood donors, 2002-2005. *Emerg. Infect. Dis.* **2008**, *14*, 436–444.
